# Superior Mesenteric Artery Syndrome: A Community Hospital Case Series

**DOI:** 10.1155/2021/4033088

**Published:** 2021-11-12

**Authors:** Brian Welch, Alex Schaal, Thomas F. O'Shea, Roberto Cantu

**Affiliations:** ^1^Flushing Hospital Medical Center, 4500 Parsons Blvd., Flushing, NY 11355, USA; ^2^Boston University School of Medicine, 72 E Concord St., Boston, MA 02118, USA

## Abstract

Superior mesenteric artery syndrome is an obstruction of the small bowel between the aorta and the superior mesenteric artery. Patients with this disease are initially managed medically and those patients who fail medical treatment require surgery. A retrospective case series of thirteen patients diagnosed with SMAS at Flushing Hospital, Flushing, NY, from 2011 to 2020 was performed. Descriptive statistics were used to summarize the characteristics of the entire cohort, and comparative statistics were used to compare the patients who failed medical treatment and required surgery to those who were successfully managed medically. Nine patients were managed conservatively and four patients required operative intervention. BMI was significantly lower in patients requiring operation compared to those who were successfully managed medically. This retrospective community hospital case series adds to the literature on SMAS and provides evidence of BMI as a potential predictor of requiring surgery in SMAS.

## 1. Introduction

Superior mesenteric artery syndrome (SMAS) is a rare gastrointestinal disorder that results from the compression of the third part of the duodenum between the angle of the aorta and superior mesenteric artery (SMA) [1]. SMAS was originally identified in 1842 by Carl von Rokitansky, and later extensively described in a comprehensive case series by David Wilkie in 1927, leading to the name “Wilkie's syndrome” [2, 3]. The normal SMA-aorta angle is from 38 to 65°, with an aortomesenteric distance (AMD) of 10-28 mm [4]. Narrowing of the angle to less than 25° with a distance less than 10 mm can cause compression of the third part of the duodenum and may cause obstructive symptoms [5, 6]. This narrowing of the SMA-aorta angle has been correlated with decreasing BMI, believed to be due to a decrease in size of the mesenteric fat pad [7]. As such, SMAS has been associated with patients that have experienced substantial weight loss, such as those with eating disorders, malignancy, AIDS, recent gastric bypass surgery, and malabsorption syndromes [8–12]. Clinically, SMAS presents with acute symptoms of postprandial epigastric abdominal pain, nausea, vomiting, fullness, and acid reflux with electrolyte imbalance [1, 2]. These symptoms overlap with many other upper GI pathologies, and therefore, SMAS is often a diagnosis of exclusion that is coupled with imaging studies to confirm the narrowing of the SMA-aorta angle [13]. SMAS is incredibly rare, with an estimated incidence of 0.013%-0.03% and therefore is often low on many clinician's differential diagnosis for these common constellation of symptoms leading to a delay in necessary treatment [14, 15]. Although this disease is often initially treated conservatively, surgical management may be warranted via a duodenojejunostomy or a Ligament of Treitz release procedure (Strong procedure) if conservative management fails to resolve symptoms [16, 17]. This case series is aimed at describing the characteristics of our patients and our surgical experience with this disease in a community hospital setting in order to add to the body of knowledge about this pathology and help identify characteristics of patients that fail conservative management.

## 2. Methods

A retrospective case series of thirteen patients diagnosed with SMAS at Flushing Hospital, Flushing, NY, from 2011 to 2020 was performed. Patient data including BMI, symptoms type/duration, associated comorbidities, SMA angle, AMD, and patient management were reviewed via our EPIC database. For the purpose of this study, patients were split into two groups: those who were managed conservatively and those who underwent either duodenojejunostomy or Strong procedure after failing conservative management. Conservative management protocols included left lateral decubitus position or knee-chest positioning, Hayes maneuver, multiple small feedings, passing of nasoenteric tube past the point of the obstruction, and intravenous fluid resuscitation. Descriptive statistics were used to summarize the data for the entire cohort; results are reported as medians and interquartile ranges or means and standard deviations, as appropriate. Categorical variables were represented as counts with percentages. Comparative statistics were used to examine differences between the conservative and operative treatment groups with unpaired two-tailed *t*-tests used to compare continuous variables and chi-square tests used to compare categorical variables.

## 3. Results

### 3.1. Demographics and Characteristics of Patients

Thirteen total patients diagnosed with SMAS were included in this study. The demographic and characteristic information for the entire cohort is shown in Table 1. The median age (IQR) at diagnosis for this cohort was 29 (24-43) years. Four patients were male, and nine were female. Median duration of symptoms (IQR) was 4 (2-180) days. The mean SMA-aorta angle was 23.75 (±10.0) degrees, the mean (±SD) AMD was 5.4 (±1.9) mm, and the mean (±SD) BMI was 21.7 (±3.1) kg/m^2^. Nine were managed conservatively (69.9%) and four patients required operative intervention (30.1%, three duodeno-jejunostomies 75% and one Strong's procedure 25%). The demographic and characteristic information for each of the two treatment modalities, along with comparative statistics, are shown in Table 2. The mean (±SD) BMI of those patients who failed conservative management and required surgery was significantly lower than those that were successfully managed with conservative therapy (18.9 ± 2.6 vs. 23.2 ± 2.3, *P* = 0.02). There was no significant difference between the two patient groups in sex, age at diagnosis, symptoms duration until diagnosis, SMA-aorta angle, and AMD.

### 3.2. Clinical Courses

The decision of which procedure to perform on the patients who failed conservative management was largely based on surgeon preference. Of note, the patient who underwent Strong's procedure had a diagnosis with Marfan's syndrome (with concurrent aortic root dilatation, severe pectus excavatum, scoliosis, and vitreous degeneration). Follow-ups were obtained from all patients. Of those operated on, 1 patient who had a duodenojejunostomy continued to have several emergency department visits (eleven times since surgery) for epigastric pain and emesis, once requiring a cholecystectomy. Several interval abdominal CT scans failed to yield a diagnosis. Eventually, she underwent an EGD which revealed duodenitis and gastritis, was treated conservatively, and has since been lost to follow up. Another patient who received a duodenojejunostomy was asymptomatic until 4 years postop when she presented with a small bowel obstruction which was treated conservatively. The other two surgically managed patients have had no issues since surgery. Of those treated conservatively, seven of them were either asymptomatic after initial management or lost to follow-up. One patient had persistently worsening reflux symptoms and was treated conservatively. One patient had undergone laparoscopic jejuno-jejunum reduction for intussusception prior to diagnosis of SMAS which was managed conservatively. She has since had two ED visits for epigastric pain/emesis—neither time surgery was consulted nor has she followed up with a general surgeon.

## 4. Discussion

SMAS is a rare condition that is well described in the literature; however, it is often misdiagnosed resulting in delay of appropriate treatment [14, 15]. This retrospective community hospital case series describes thirteen patients with imaging confirmed diagnoses of SMAS with symptomatic presentations that were treated with either conservatively or operatively. This series is aimed at adding to the literature on SMAS, specifically in the community hospital setting, aiding in syndrome's accurate diagnosis, and providing insight into potential characteristics of patients that fail conservative management.

Radiological criteria used in the diagnosis of patients with SMAS include dilatation of the first and second parts of the duodenum, sudden vertical compression of the mucosal fold, flow of the barium against the peristaltic flow in the area proximal to the obstruction, delay of 4-6 hours in the gastroduodenal region, or relief of the obstruction when the patient was repositioned in the left lateral decubitus/knee-chest positions [13, 17]. The most sensitive finding for SMAS on imaging, however, is a SMA-aorta angle of <25 degrees with an AMD of <8 mm [4, 5]. In our cohort, the mean SMA-aorta angle was 23.75 (±10.0) degrees, and the mean (±SD) AMD was 5.4 (±1.9) mm in patients presenting with symptoms of SMAS (Table 1) matching the recommended diagnostic guidelines. However, there were still three patients who presented symptomatically with SMA − aorta angles > 25 degrees; therefore, our data suggest that SMA-aorta angle is not a completely sensitive test for SMAS, and it should still be considered as a potential cause of obstructive symptoms even in the presence of a larger SMA-aorta angle with a less than 8 mm AMD.

Our cohort had a median age at diagnosis of 29 with IQR 24-43, and more female patients were diagnosed than male patients. Our patients exhibited a wide range of symptom duration before diagnosis of SMAS, with some experiencing symptoms for as long as 5 years and some as little as 2 days before diagnosis. Most common symptoms reported were epigastric pain, nausea, vomiting, and fullness. There was no significant difference in the duration of symptoms before diagnosis between patients that were managed conservatively compared to those with surgery.

Management of SMAS can vary depending on the severity of the symptoms. A nonoperative approach is typically the first line of treatment [16]. The three most common operative treatments are gastrojejunostomy, duodenojejunostomy, and Strong's operation (mobilization of the right colon then sectioning of the ligament of Treitz with mobilization of the transverse and ascending duodenum) [13]. Nine of our patients were successfully managed with strategies including left lateral decubitus position or knee-chest positioning, Hayes maneuver, multiple small feedings, passing of nasoenteric tube past the point of the obstruction, and intravenous fluid resuscitation. Four of our patients failed this conservative management regiment and required operative intervention. The BMI of the patients who failed conservative treatment was found to be significantly lower than those that were managed successfully with conservative treatment. In this cohort, there was no other significant differences between the treatment groups. This finding is consistent with previous data that demonstrate a correlation between decreased BMI and decreased SMA aorta angle [7]. This suggests a role for BMI as a predictor of which patients will require surgical management; however, due to the low power nature of this case series, further larger scale studies are warranted. But given the rarity of this disease, it may be hard to establish.

There are several limitations of this study. First, this case series has a very low sample size of 13 patients. Part of this is inherent in studying SMAS due to its low incidence; thus, multicenter studies should be conducted to provide further significant data. Second, our postdiagnosis data is underwhelming due to many patients in both treatment cohorts who were lost to follow-up; however, the patients that were managed conservatively and surgically seemed to have unique posttreatment complications and courses. Finally, it is important to note that the decision to perform surgery, and which specific operation was performed, was at the preference of the attending surgeons.

SMAS is a disease that requires consideration for any patient with obstructive GI symptoms, especially ones with lower BMI or other predisposing conditions. This retrospective community hospital case series adds to the literature on SMAS, specifically in the community hospital setting, and provides evidence of BMI as a potential predictor for failure of conservative management that requires further investigation.

## Figures and Tables

**Table 1:** Characteristics of patients with SMAS.

Characteristic	Patients (*N* = 13)
Patient age at diagnosis (years), median (IQR)	29 (24-43)
Patient sex, *N* (%)	
(i) Male	4 (30.1%)
(ii) Female	9 (69.9%)
Symptoms duration (days), median (IQR)	4 (2-180)
BMI, mean (SD)	21.7 (3.1)
SMA-aorta angle (degrees), mean (SD)	23.8 (10.0)
Aortic mesenteric distance (millimeter), mean (SD)	5.4 (1.9)
Treatment, *N* (%)	
(i) Conservative treatment	9 (69.9%)
(ii) Operative treatment	4 (30.1%)
(1) Duodenojejunostomy	3 (75%)
(2) Strong procedure	1 (25%)

**Table 2:** Characteristics of conservatively treated vs. operatively treated patients.

Characteristics	Conservative Tx (*N* = 9)	Operative Tx (*N* = 4)	*P* value
Patient sex, *N* (%)			
(i) Male	4 (44.4%)	0 (0.0%)	0.11
(ii) Female	5 (66.6%)	4 (100%)	
Patient age at diagnosis (years), mean (SD)	31.4 (12.9)	33.25 (20.7)	0.85
Symptom duration until diagnosis (days), mean (SD)	274.89 (594.17)	185.25 (363.17)	0.78
BMI, mean (SD)	23.2 (2.3)	18.9 (2.6)	**0.02**
SMA-aorta angle (degrees), mean (SD)	(32.1-35)	34 (32.6-34.5)	0.97
Aortic mesenteric distance (mm), mean (SD)	5.7 (2.0)	4.8 (1.5)	0.43

## Data Availability

The patient data used to support the findings of this study are available from the corresponding author upon request.
